# Dual agarose magnetic (DAM) ChIP

**DOI:** 10.1186/1756-0500-2-250

**Published:** 2009-12-14

**Authors:** Lata Balakrishnan, Barry Milavetz

**Affiliations:** 1 Department of Biochemistry and Molecular Biology, University of North Dakota, Grand Forks, ND, 58203, USA; 2 Department of Biochemistry and Biophysics, University of Rochester, Rochester, NY, 14642, USA

## Abstract

**Background:**

Chromatin immunoprecipitation (ChIP) has become a very popular technique to study epigenetic regulation because it can be used to identify proteins and protein modifications present at specific locations in chromatin. While techniques have been developed to investigate epigenetic modifications present in chromatin during a specific biological function such as transcription, they depend upon the ability of the ChIP to analyze two epitopes on the same chromatin and are generally time consuming, difficult to perform, and not very sensitive. The Dual Agarose Magnetic (DAM) ChIP procedure described here is designed to address these shortcomings.

**Findings:**

Protein A agarose and protein G magnetic beads bound with different IgGs have been combined in a single Chromatin Immunoprecipitation (ChIP) assay to analyze for the presence of two epitopes on the same chromatin at the same time. This procedure has been used with non-immune rabbit IgG bound to either the agarose or beads in order to include an internal negative control for non-specific binding of chromatin. The procedure has also been used with various antibodies including those targeting RNA Polymerase II and replication protein A 70 to determine whether specific forms of modified histones are present in either transcribing or replicating forms of SV40 minichromosomes respectively.

**Conclusions:**

The DAM ChIP procedure is a rapid, simple, and sensitive technique to characterize two epitopes located in the same chromatin. It should be particularly useful for the rapid screening of epigenetic modifications present in biologically active chromatin.

## Findings

Chromatin Immunoprecipitation (ChIP) has become an extremely powerful tool for the characterization of biologically functional chromatin. Specific antibodies bound to either protein A agarose or protein G magnetic beads are used to immune-select fragments of chromatin containing the target epitope of the antibody followed by PCR amplification to identify and quantitate the DNA present in the chromatin [1-4]. A carrier like agarose or magnetic beads is required in both of these procedures because it is necessary to separate the chromatin bound to antibody from contaminating chromatin during purifications. In the standard procedure this is done by sedimenting the agarose by low speed centrifugation, while in the modification with magnetic beads a magnet is used to bind the beads.

**Figure 1:**
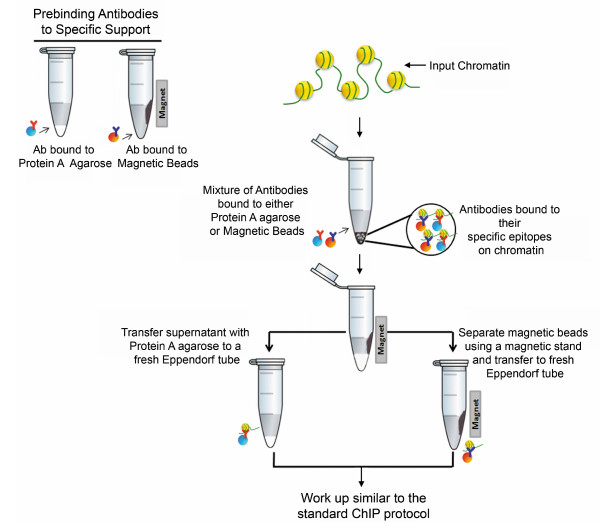
**Schematic for Dual Agarose Magnetic (DAM) ChIP protocol**.

We have taken advantage of the distinct properties of agarose and magnetic beads and developed a procedure which allows chromatin immunoprecipitation with two antibodies present in the same tube. A schematic representation of this procedure is shown in Figure 1. One of the antibodies is bound to agarose, while the second antibody is bound to magnetic beads. Following binding of the chromatin to one of the antibodies and addition of the other antibody, the agarose and magnetic beads are separated based upon their difference in magnetic properties.

We first determined whether normal rabbit IgG and antibody to hyperacetylated histone H4 (Hyp H4) when bound to different carriers in the same ChIP would still function as they do in typical ChIP assays. We have extensively studied the chromatin structure of SV40 minichromosomes and showed that they contain hyperacetylated histones [5-7]. All ChIPs were performed using a Millipore ChIP assay kit (17-295) with binding and washing conditions as previously described [5-7]. Antibody to Hyp H4 and normal rabbit IgG were incubated in 750 μl of ChIP dilution buffer (CDB) and either 80 μl of protein A agarose or 25 μl of protein G magnetic beads for from 4 hours. Following the binding step, unbound serum constituents were removed from the agarose and magnetic beads by low speed centrifugation and interaction with a magnet in a stand (Promega; Z5342), respectively. The supernatants were removed and the agarose and magnetic beads resuspended in 100 μl of ChIP dilution buffer.

For standard ChIPs the resuspended agarose and magnetic beads carrying either IgG or antibody to Hyp H4 were added to 650 μl of CDB in an Eppendorf tube. Purified SV40 minichromosomes [5-7] {75 μl} were added and the assay was incubated at 4°C with constant end over end rotation over night. The chromatins bound to agarose and magnetic beads were purified, eluted from each carrier with two 15 minute incubations using the SDS lysis buffer in the Millipore kit, and analyzed as previously described [5-7].

**Figure 2:**
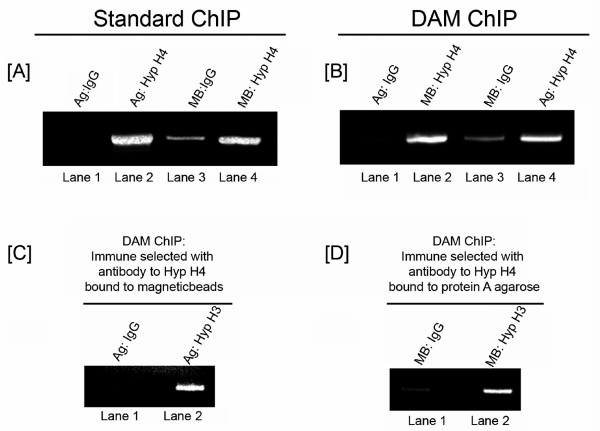
**DAM ChIPs**. 48 hour unfixed SV40 minichromosomes were analyzed by (a) standard (Std ChIP) or (b, c, d) DAM chromatin immunoprecipitation (DAM ChIP) and the immunoprecipitates were amplified by PCR (32 cycles) with primer sets to the early region of the SV40 genome as previously described [5-7]. (a) Std ChIPs with antibodies bound to protein A agarose or protein G magnetic beads. Lane 1, Std ChIP with IgG (7.5 μl) (Bethyl Laboratories; P120-101) bound to agarose (Millipore, 17-295); lane 2, Std ChIP with Hyp H4 (7.5 μl) (Millipore; 06-866) bound to agarose; lane 3, Std ChIP with IgG (7.5 μl) bound to magnetic beads (Active Motif, 53014); lane 4, Std ChIP with Hyp H4 (7.5 μl) bound to magnetic beads. Ag; Agarose, MB; magnetic beads. (b) DAM ChIPs in which chromatin was analyzed with IgG (7.5 μl) bound to agarose (Lane 1) and Hyp H4 (7.5 μl) bound to magnetic beads (Lane 2) in the same tube or IgG (7.5 μl) bound to magnetic beads (Lane 3) and Hyp H4 (7.5 μl) bound to agarose (Lane 4) in the same tube. (c) DAM ChIPs immune selected with antibody to Hyp H4 bound to magnetic beads. Purified immune selected chromatin was divided into two aliquots and an aliquot was added to a tube containing either IgG or Hyp H3 bound to agarose. Lane 1, DAM ChIP with IgG (7.5 μl); lane 2, DAM ChIP with Hyp H3 (7.5 μl) (Millipore; 06-599). DAM ChIPs immune selected with antibody to Hyp H4 bound to agarose. Purified immune selected chromatin was divided into two aliquots and an aliquot was added to a tube containing either IgG or Hyp H3 bound to magnetic beads. Lane 1, DAM ChIP with IgG (7.5 μl); lane 2, DAM ChIP with Hyp H3 (7.5 μl).

As expected from our previous studies [5-7], there was very little if any PCR amplification product in the samples from chromatin bound to IgG (Figure 2A; lanes 1 and 3) and significant product formation in samples from chromatin bound to Hyp H4 (lanes 2 and 4).

For DAM ChIPs 100 μl of agarose containing IgG and 100 μl of magnetic beads containing Hyp H4 were added to an Eppendorf tube containing 550 μl of CDB. Similarly, in a separate tube 100 μl of magnetic beads containing IgG and 100 μl of agarose containing Hyp H4 were added to 550 μl of CDB. SV40 minichromosomes {150 μl} were added to each of the tubes and the assay incubated as above. Following incubation overnight, the agarose and magnetic beads were separated. The tubes were placed adjacent to a magnet stand for 2 minutes. The supernatant including the agarose was then removed and transferred to a new tube also in the magnet stand. The magnetic beads were washed once with 100 μl of CDB and the wash liquid added to the new tube. Following a further 2 minutes to remove any remaining magnetic beads the supernatant containing the agarose was again transferred to a new tube. The agarose was sedimented by low speed centrifugation and the liquid removed. The tubes containing magnetic beads and agarose were then washed and the chromatin eluted and prepared as described above.

The results of this analysis are shown in Figure 2B. Again we observed very little amplification of DNA present in IgG bound chromatin (lanes 1 and 3) and extensive amplification of the DNA present in the Hyp H4 bound chromatin (lanes 2 and 4). The similarity in results between Figure 2A indicate that both the agarose and magnetic beads carrying IgG or antibody function together in the DAM ChIP assay the same as when they are separate in a standard ChIP assay. It is not clear why there is more amplification with the IgG bound to magnetic beads than agarose.

**Figure 3:**
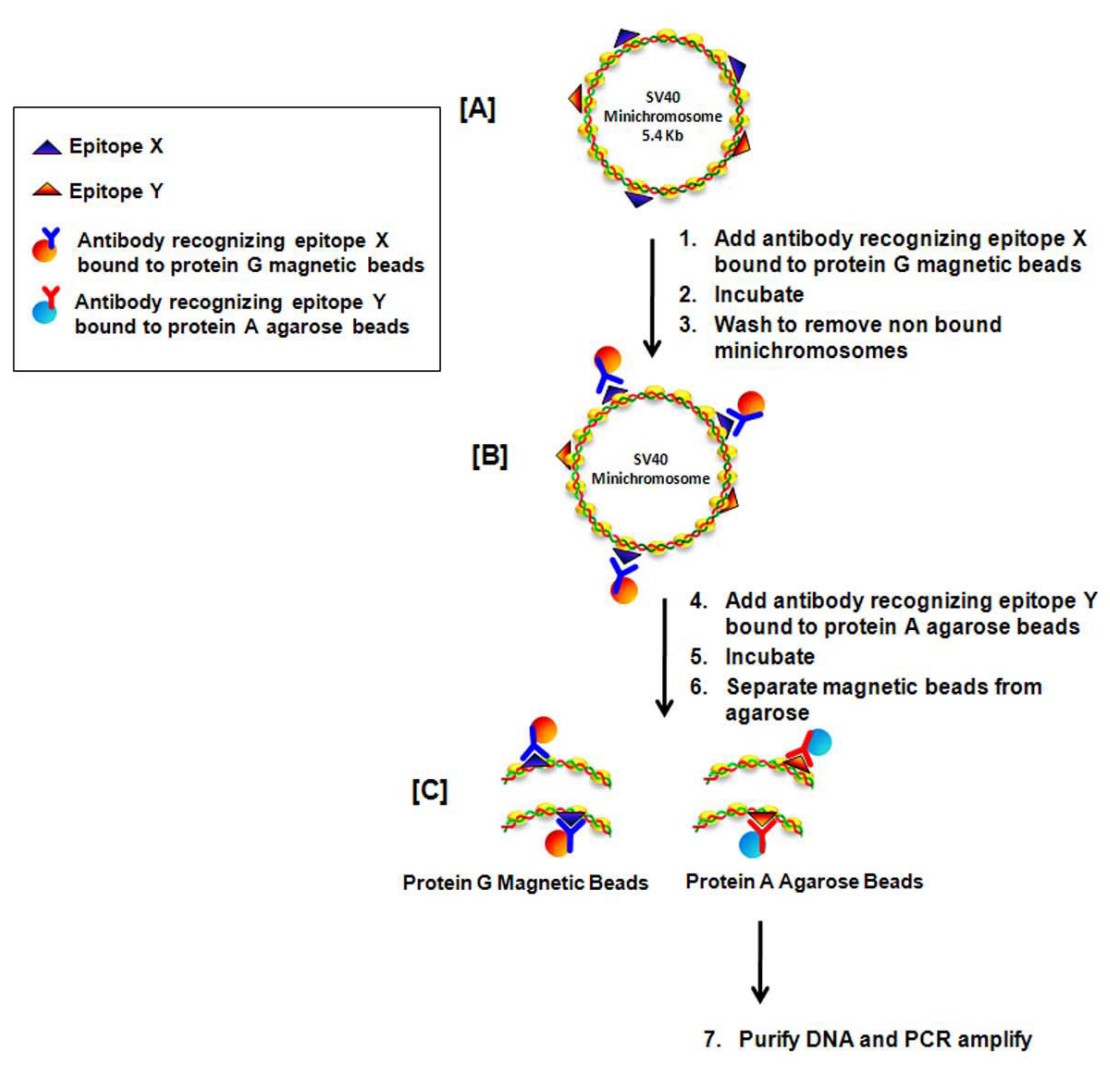
**Schematic representation of the strategy used for DAM ChIP analysis of protein modifications in immune selected SV40 minichromosomes**.

Next, we investigated whether DAM ChIPs could be used to determine whether two different proteins or protein modifications can be observed in the same SV40 minichromosomes. The DAM ChIP strategy that we used to demonstrate this is shown in Figure 3. An SV40 minichromosome containing two epitopes of interest, X and Y, is shown in part A. While the schematic will be described using an antibody to protein X bound to the magnetic beads and an antibody to protein Y bound to agarose, the DAM ChIP can also be performed with the antibody to protein X bound to the agarose beads and an antibody to protein Y bound to magnetic beads.

In the first step, a pool of SV40 minichromosomes isolated from infected cells was immune selected with antibody to X bound to magnetic beads and the magnetic beads containing the selected minichromosomes washed according to the Millipore protocol to remove the contaminating SV40 minichromosomes lacking this epitope (Part B). In the second step antibody to Y bound to agarose was added and incubated overnight with end over end rotation. We hypothesized that if a minichromosome bound to the magnetic beads also contained the protein target for the second antibody in an accessible location, that portion of the fragment containing the antibody target would be bound by the antibody on the second carrier and would ultimately be transferred to it as a result of shearing of the chromatin (Part C). Following the incubation, the magnetic beads were separated from the agarose and the agarose was subsequently washed according to the Millipore protocol. The bound chromatin was eluted from the agarose using the Millipore lysis buffer and prepared for PCR analysis.

Typical results of this analysis are also shown in Figure 2C and 2D. Antibody to Hyp H4 was either bound to magnetic beads for the first step (C) or agarose (D). Normal IgG or antibody to hyperacetylated histone H3 (Hyp H3) bound to either agarose or magnetic beads was used as the second antibody. We used IgG as a negative control for non-specific binding and antibody to Hyp H3 as a positive control since we have already shown that Hyp H4 and Hyp H3 can be found together on SV40 minichromosomes [5,8,9]. As shown in this figure we observed a robust amplification product from the chromatin present on the carriers with antibody to Hyp H3 (lane 2, C and D) and very little amplification product from any chromatin bound to IgG (lane 1 C and D) regardless of which carrier was used in the first and second steps indicating that both Hyp H4 and Hyp H3 were present in the same SV40 minichromosomes using the DAM procedure.

**Figure 4:**
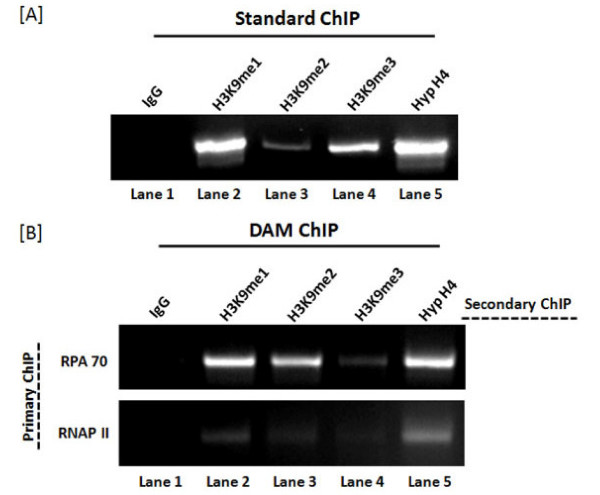
**DAM ChIP analysis of replicating and transcribing SV40 minichromosomes**. 48 hour unfixed SV40 minichromosomes were analyzed by [a] standard chromatin immunoprecipitation using either IgG, or antibodies to H3K9me1, H3K9me2, H3K9me3, or Hyp H4 (7.5 μl each) bound to protein A agarose or [b] DAM ChIPs using antibody bound to magnetic beads in the first step which recognized RPA70 (SCBT; sc-25376) (50 μl) to immune select replicating minichromosomes or antibody which recognized RNAPII (SCBT; sc-900) (50 μl) to immune select transcribing minichromosomes. Following immune selection the magnetic beads were washed according to protocol and then divided into five equal aliqouts with an aliquot added to a tube which contained either IgG, or antibodies to H3K9me1, H3K9me2, H3K9me3, or Hyp H4 (7.5 μl each) bound to agarose. The immunoprecipitates bound to agarose from both analyses were then amplified by PCR (a: 32 cycles or b: 45 cycles) with primer sets to the early region of the SV40 genome as previously described [5-7]. Lane 1, ChIP with IgG; lane 2, ChIP with H3K9me1 (Abcam; ab9045); lane 3, ChIP with H3K9me2 (Abcam; ab1220); lane 4, ChIP with H3K9me3 (Abcam; ab8898); lane 5, ChIP with Hyp H4.

Finally, we analyzed replicating and transcribing SV40 minichromosomes for the presence of modified histones (Figure 4). Using ChIP validated antibodies to replication protein A 70 (RPA70) [7] and RNA Polymerase II (RNAPII) [9] to immune select replicating and transcribing SV40 full length minichromosomes respectively, we analyzed the minichromosomes for the presence of histone H3 mono-methyl lysine 9 (H3K9me1), histone H3 di-methyl lysine 9 (H3K9me2), histone H3 tri-methyl lysine 9 (H3K9me3) and Hyp H4 with the corresponding antibodies. All these antibodies except for H3K9me2 which was a mouse monoclonal antibody were rabbit polyclonal antibodies. In standard ChIPs all of these antibodies recognize SV40 minichromosomes isolated at this time and yield robust amplification products (Figure 4A, lanes 2 through 5). When replicating minichromosomes (Figure 4B, RPA70 Primary ChIP) were analyzed, we observed robust amplification products from the DAM ChIPs with antibody to H3K9me1 (lane 2), H3K9me2 (lane 3), and Hyp H4 (lane 5), but not from the DAM ChIPs with the negative control IgG (lane 1) or antibody to H3K9me3 (lane 4). When transcribing minichromosomes (Figure 4B, RNAPII Primary ChIP) were analyzed, we observed a weak but definite amplification product in the Hyp H4 DAM ChIP (lane 5) and very weak amplification products from all of the other DAM ChIPs. These results indicated that replicating minichromosomes contained Hyp H4 as previously reported [7], H3K9me1, and H3K9me2 but not H3K9me3. In contrast transcribing minichromosomes appeared to contain Hyp H4 as previously observed [9] and little if any of the methylated forms of H3K9. The lack of methylated H3K9 is not surprising since this modification is thought to be repressive [10].

We have demonstrated the feasibility of using two antibodies which are individually bound to different carriers in a single ChIP assay and present evidence that this strategy can be used either to include an internal negative control during an assay or to determine whether two proteins or protein modifications can be located in a large fragment of chromatin. The advantages of the former procedure are that only half as much input chromatin is required, the washes can be performed while the two carriers are together, and the binding conditions for the negative control and test antibody are identical. The major advantage of DAM ChIPs is its simplicity. The procedure is relatively rapid and unlike most other procedures like ReChIP [11] does not require an elution step. After preparing our manuscript we became aware of a similar technique which was independently developed in another laboratory at the same time [12]. While the basic concept of using two different carriers for antibodies is similar in both our procedure and the procedure from the Medeiros group [12], we believe the two procedures differ significantly. Conceptually the Medeiros procedure is designed as a true sequential ChIP which utilizes the high affinity of the biotin-streptavidin interaction in conjunction with short incubation times and fragmented chromatin to allow the magnetic beads containing biotin conjugated antibody to bind more tightly to the chromatin present on agarose than the antibody originally present on the agarose. Thus, the authors report that they observe direct interactions between the magnetic beads and agarose and that the chromatin is essentially transferred from the agarose to the magnetic beads during the procedure. Our procedure is not a true sequential ChIP. Instead, it is based upon the concept that a large fragment of chromatin bound at two distant sites by different antibodies present on carriers will be sheared due to rotation during the relatively long incubation into smaller fragments bound to each of the antibodies. For this reason we have never observed direct interactions between the magnetic beads and agarose during ChIPs. The two procedures also differ in the following ways: (i) Our procedure does not require biotin conjugated antibodies for ChIPs since the antibodies used in the DAM ChIP are bound directly to Protein A agarose or Protein G magnetic beads; (ii) The DAM ChIP protocol looks at protein binding over a much larger sequence (~5000 bp) whereas the technique described by Medeiros *et al *primarily looks at proteins bound to promoter sequences, which are smaller in size (1000 bp or less); (iii) Extensive sonication of chromatin is not required in the DAM ChIP procedure; (iv) Finally, we show similar results using the DAM ChIP technique regardless of which carrier is used for the first or second antibody. Using this technique we also observed no cross contamination with either of the carriers. Considering the significant advantages of the DAM ChIP protocol we anticipate this modified technique will be a useful tool for the study of epigenetic modification.

## Competing interests

The authors declare that they have no competing interests.

## Authors' contributions

LB and BM contributed equally to this work in the design and execution of experiments, and the preparation of the manuscript.
